# The Interaction Effect of Parental Rejection and Oxytocin Receptor Gene Polymorphism on Depression: A Cross-Cultural Study in Non-Clinical Samples

**DOI:** 10.3390/ijerph19095566

**Published:** 2022-05-04

**Authors:** Vincenzo Paolo Senese, Kazuyuki Shinohara, Paola Venuti, Marc H. Bornstein, Vittorio Rosanio, Carla Nasti, Michelle Jin-Yee Neoh, Marzia Maresca, Gianluca Esposito

**Affiliations:** 1 Psychometric Laboratory, Department of Psychology, University of Campania “Luigi Vanvitelli”, 81100 Caserta, Italy; vittorio.rosanio@outlook.it (V.R.); carla91nasti@gmail.com (C.N.); 2 Department of Neurobiology and Behavior Unit of Basic Medical Sciences Course of Medical and Dental Sciences, Nagasaki University Graduate School of Biomedical Sciences, Nagasaki 852-8042, Japan; kazuyuki@nagasaki-u.ac.jp; 3 Department of Psychology and Cognitive Sciences, University of Trento, 38100 Trento, Italy; paola.venuti@unitn.it (P.V.); gesposito79@gmail.com (G.E.); 4 Eunice Kennedy Shriver National Institute of Child Health and Human Development, Bethesda, MD 20810, USA; marc.h.bornstein@gmail.com; 5 Psychology Program, School of Social Sciences, Nanyang Technological University, Singapore 637511, Singapore; michelle008@e.ntu.edu.sg; 6 Institute of Relational and Family Psychology and Psychotherapy (ISPPREF), 80127 Napoli, Italy; marzia.maresca@gmail.com

**Keywords:** depression, gene–environment interactions, oxytocin, interpersonal acceptance–rejection theory, psychological adjustment

## Abstract

Parental rejection has been consistently empirically implicated in a wide array of developmental, behavioural and psychological problems worldwide. However, the interaction effect between parental rejection in childhood and the oxytocin receptor genotype on psychological adjustment has yet to be investigated. The present study aimed to investigate gene–environment interaction effects between parental rejection (maternal and paternal) and oxytocin receptor (OXTR) gene polymorphisms (rs53576 and rs2254298) on depressive symptoms in adults in different cultural contexts. Adults from Italy and Japan (*N* = 133, age = 18–27 years, females = 68) were preliminarily genotyped and then completed the Parental Acceptance-Rejection Questionnaire for mothers and fathers and the Beck Depression Inventory. Hierarchical multiple regression analysis showed that paternal rejection was related to self-reported depression and that the effect of parental rejection was moderated by OXTR gene polymorphisms and nationality. Among Italians, OXTR rs2254298 A-carriers showed resilience to negative early parental care, whereas among Japanese, OXTR rs53576 non-A-carriers showed resistance to negative early paternal care. These findings align with expected relations between perceived acceptance–rejection and an individual’s psychological adjustment, as proposed by interpersonal acceptance–rejection theory, and indicate the need for future studies adopting a multicultural and multilevel approach to better understand how the effects of parental rejection extend into adulthood.

## 1. Introduction

Adverse childhood experiences and early life stress are common societal problems worldwide [1] and have been consistently shown to be associated with mental health problems across the lifespan in different populations. Children with adverse childhood experiences are more likely to develop mental health problems such as anxiety, depression [2], post-traumatic stress disorder [3] and substance use problems [4]. Parental acceptance–rejection makes up the warmth dimension of parenting [5,6,7], where parental rejection refers to the absence or withdrawal of parental love and the presence of physically, psychologically and symbolically hurtful behaviours and affect toward children [8]. Similarly, parental rejection has been shown to be a predictor of psychological and behavioural problems, and there is an immense body of cross-cultural and intra-cultural research supporting the link between parental rejection and depressive symptoms in children; see [9] for a meta-analysis and [10] for a review. The well-documented empirical association of parental rejection with psychopathology is a clear indication of the importance of understanding factors that influence the relationship between parental rejection and depression.

### 1.1. Parental Rejection and Depression

Interpersonal acceptance–rejection theory (IPARTheory; [5,11,12,13]) is composed of three sub-theories and attempts to predict and explain the consequences and correlates of interpersonal acceptance and rejection extending from childhood to adulthood. A core premise of the theory proposes that acceptance or rejection by an intimate partner has a major influence on an adult’s personality and personality adjustment. IPARTheory is an evidence-based theory grounded upon research indicating the universal relation between parental acceptance–rejection and subsequent psychological adjustment in both children and adults [12,14,15]. Firstly, parental rejection, a commonly cited environmental risk factor for depression, has been implicated in both clinical depression and depressed affect across countries such as Australia [16], China [17] and Italy [18] and within almost every major ethnic group in the United States, including Asian Americans [19], African Americans [20] and Mexican Americans [21]. Secondly, longitudinal studies indicate that perceived parental rejection in childhood predicts the development of depressive symptoms in adolescence and adulthood [22,23]. Within this large body of research, it is clear that there is an apparent convergence towards the same conclusion that parental rejection is associated with the development of depressive symptoms.

While the link between parental rejection and psychological adjustment is well supported, a meta-analysis revealed that parental rejection only accounts for 26% of the variability in children’s psychological adjustment and 21% of the variability in adults [24]. This indicates that a substantial proportion of variance remains to be explained by a multitude of other factors, including but not limited to genetic, cultural, behavioural and neurobiological ones [25,26,27]. Hence, the present study aims to expand on IPARTheory and these empirical findings by investigating not only the role of parental rejection (maternal and paternal) in depression but also the moderating effect of genetics and culture on this association.

### 1.2. Oxytocin, Childhood Experience and Depression

Behaviour genetics studies have revealed the role of genetics in depression, with heritability estimates ranging between 40–70% [28], although no single genes have been identified yet. Additionally, studies documenting gene–environment interactions have begun to demonstrate that individuals tend to inherit genetic predispositions for a particular disorder, rather than inheriting any particular disorder itself (see, for example, [29]). In a landmark study by Caspi et al. [30], a gene–environment interaction was found in which polymorphisms in the serotonin transporter gene (5-HTT) promoter moderated the influence of stressful life events on depression. Another candidate gene for depression is the oxytocin receptor (OXTR) gene. The oxytocin system has been implicated in complex social behaviours such as affiliative behaviour, relationships with romantic partners and friends and the regulation of the stress response (see [31] for a review). Results from a number of studies suggest that oxytocin may serve a protective function as an anti-stress hormone by enhancing social behaviours and the ability to draw comfort from social contacts [32,33]. In terms of human research on the link between OXTR and psychopathology, both family and population studies have linked several OXTR single-nucleotide polymorphisms (SNPs)—variants in alleles at a particular gene locus—with psychopathology involving social dysfunction, including depression. In particular, the role of OXTR SNPs rs53576 (G/A) and rs2254298 (G/A) have been heavily underscored.

Firstly, G allele homozygotes for rs53576 have been found to be associated with several positive prosocial characteristics, including (i) greater empathy [34], (ii) higher self-esteem and optimism [35], (iii) general sociality as rated by peers [36] and (iv) lower cortisol levels during the Trier Stress Test [37]. The presence of an A allele in rs53576 has also been associated with higher negative affect in non-clinical populations [35]. G allele homozygotes also displayed higher levels of positive affect and resilient coping when brought up in warm or stable family environments, which was not observed in A carriers [38]. However, contrary to these findings, for G allele homozygotes, other studies have also revealed: (i) greater adult emotion dysregulation [39], which has been implicated in depression, in individuals who experienced severe childhood maltreatment, (ii) a positive association with unipolar depression in an Italian sample [40] and (iii) a positive correlation between childhood maltreatment and the severity of depression symptoms, indicating a possible gene–environment interaction between early childhood experience and OXTR genotypes [41].

Secondly, the A allele of OXTR rs2254298 has been suggested to be involved in plasticity processes, which increases resilience in dealing with stressful environments, especially those occurring during early life [42] that often play a role in susceptibility to depression in individuals. However, similar to rs53576, the presence of an A allele for rs2254298 has been associated with higher negative affect in non-clinical populations [43,44], and the homozygous GG genotype for rs2254298 showed a positive association with unipolar depression [40].

Notably, some of these findings point towards the interaction of adverse early life experiences with oxytocin and the possibility that OXTR genotypes could influence sensitivities to both positive and negative environments [38,41,42,45]. Given that adverse early life experiences and stress, such as abuse and neglect, in combination with OXTR genotypes, are associated with an increased risk of depression [46], these findings further strengthen the argument for oxytocin as a candidate gene related to depression. In addition, changes to oxytocin levels and reactivity due to adverse early life experiences have also been observed in a number of studies. Firstly, early adverse social experiences alter the OXT system, enhancing individual vulnerability to the pathologic effect of stress [47]. Secondly, oxytocin levels in cerebrospinal fluid (CSF) were reduced in adult women with a history of childhood abuse, with progressively lower levels observed as the number of types of maltreatment increased [48]. Similarly, lower plasma oxytocin concentrations were observed in men who reported higher levels of early life adversity and depressive scores [49]. Thirdly, higher oxytocin levels were observed in individuals with severe childhood maltreatment compared to those who experienced less severe forms of childhood maltreatment in a Japanese sample [50].

In summary, while oxytocin appears to be involved in processes often implicated in depression and there is some evidence supporting the association between the two, there has also been evidence to the contrary and even findings indicating a lack of relationship between the two [51]. It has been suggested that such contradictory evidence in genetic studies may be attributable to the failure to account for environmental influences or interactive effects between genetics and the environment or other gene loci [52]. A recent review also found significant involvement of rs53576 and rs2254298 in gene–environment interactions, with early parental care modulating the risk for a range of psychopathologies, including depression [53], suggesting that the interaction between parental rejection and OXTR genotype likely has an effect on depression. Moreover, the diathesis–stress hypothesis [54] is also supported by evidence showing an interaction effect between OXTR (rs6770632) and poor parenting style on depressive symptoms in young adults, with A allele homozygotes reporting higher levels of depressive symptoms than C carriers [55]. Hence, the present study aims to investigate the influence of OXTR rs53576 and rs2254298 genotypes on depression in the context of parental rejection in childhood by examining gene–environment interactions.

### 1.3. Oxytocin Receptor Polymorphisms across Countries

Although the reason is not well-understood, the effects of OXTR genotypes may be moderated by ethnicity and culture [56], which could possibly be another explanation accounting for the contradicting evidence in genetic studies on OXTR. The gene–culture coevolution theory proposes that novel environments are created under different cultures, where genetic selection operates and selects for different cognitive and neural architectures that facilitate the transmission of particular cultural values [57]. Support for this theory can be observed from evidence of differing allelic frequencies of the OXTR gene across countries [58], where (i) allele frequencies and linkage disequilibrium patterns differ between Asians and Caucasians [59], and (ii) the A allele of rs53576 and rs2254298 is more common in Asia than in Europe [56]. Luo and Han [58] found that there was an association between collectivistic cultural values and A allelic frequency of rs53576, where there are more A allele carriers in nations with more strongly dominating collectivistic cultural values. In addition, Luo and Han [58] found that the presence of the A allele was predictive of the prevalence of major depressive disorder across nations, where this association was mediated by collectivistic cultural values. These findings lay the foundation for the expectation of differential genetic influences across cultures on neural activity guiding human behaviour. For example, Chiao and Blizinsky [60] suggested that cultural values may function in fine-tuning social behaviour in order to reduce environmental risk factors, while gene frequency plays a crucial role in explaining the adoption of different cultural norms across the world.

Similarly, a gene–culture interaction model has been developed to explain how genetic and sociocultural factors interact in shaping psychological tendencies and behaviours at an individual level [61]. It posits that genes provide a basis for susceptibility to cultural environments [45,62] and influence an individual’s engagement in cultural-specific behaviours [61]. For example, the gene–culture interaction has been observed between OXTR rs53576 and emotional processes and emotion-related behavioural tendencies. Firstly, there were differences in the phenotype of G allele homozygotes in terms of emotion suppression between Americans and Koreans. Among Americans, G allele homozygotes reported less emotion suppression compared to A allele homozygotes, whereas the opposite trend was observed in Koreans, where G allele homozygotes reported more emotion suppression compared to A allele homozygotes [63]. Secondly, similar gene–culture interactions were observed in terms of emotional support-seeking behaviour. In conditions of great distress, G allele carriers among Americans sought more emotional support from others compared to A allele homozygotes, whereas no such genetic differences in emotional support-seeking behaviour were observed in Koreans [64]. Interestingly, however, Koreans exposed to American culture showed a similar pattern of emotional support-seeking behaviour to Americans [64].

Another gene–culture interaction was also observed in a study by Sasaki, Kim and Xu [65] on the benefits of religiosity on psychological well-being. The study found that among Koreans, G allele homozygotes for rs53576 experienced a positive correlation between psychological well-being and religiosity, where higher psychological well-being was observed with greater religiosity. However, among Americans, G allele homozygotes experienced lower psychological well-being if they were more religious. These findings indicate a gene–culture interaction moderating the association between religiosity and psychological well-being. They also suggest that the beneficial effects of religion on well-being observed in those with a genetic predisposition for social sensitivity may be dependent on adequate opportunities for social affiliation, provided as a function of their cultural context [65].

Hence, based on differences in allelic frequencies between different ethnicities and regions, along with these initial findings on gene–culture interactions, the present study aims to investigate possible gene–culture interactions in depression and whether culture moderates the gene–environment interaction effects of parental rejection and OXTR genotypes on depression.

### 1.4. Present Study

Building upon IPARTheory and previous findings on OXTR genotype and depression, the present study aims to investigate the gene–environment interactions between parental rejection and OXTR SNPs (rs53576 and rs2254298) and their effects on self-reported depression in adults in different cultural contexts. Firstly, in line with IPARTheory and findings indicating gene–environment interaction effects between OXTR genotypes and early parental care on the risk of psychopathology [53], we hypothesise that there are significant gene–environment interaction effects between parental rejection and OXTR SNP rs53576 and rs2254298 genotypes on depression in adults. In particular, we expect that OXTR polymorphisms may moderate the association between perceived parental rejection and the level of depression. Secondly, based on previous findings on gene–culture interactions, we hypothesise that these gene–environment interactions are moderated by the ethnicity of the individual. In particular, in line with the literature, we expect that the moderation effect across the two cultures is regulated by different polymorphisms. The interaction effects were also analysed to determine if they are more congruent with the diathesis–stress hypothesis [54], which assumes that some genetic polymorphisms can act as risk factors when associated with a negative environment, or the differential susceptibility hypothesis [45], which assumes that genetic polymorphisms could be considered environmental sensitivity factors that amplify the effect of the environment, either positively or negatively.

## 2. Method

### 2.1. Participants

A total of 133 participants (female = 68, male = 65; *M* age = 21.2 years, *SD* = 2.6), ranging from 18–27 years old, were recruited for the study (see Table 1). Participants were sampled in two different countries: Italy (*n* = 78; female = 39, male = 39; *M* age = 22.5 years, *SD* = 2.2) and Japan (*n* = 55; female = 29, male = 26; *M* age = 19.4 years, *SD* = 1.9). The educational level of the participants ranged from middle school to college. All participants were tested individually.

### 2.2. Procedure

The study was approved by the Ethics Committee in the Department of the first author and was conducted in conformity with the Helsinki Declaration. In both populations, participants were recruited by quota sampling, which is a non-probability sampling method that was used to create equivalent and balanced samples across the two populations. To be included in the sample, participants had to be over 18 and not be a parent or diagnosed with a psychopathological disorder. As shown in Table 1, no participants with “severe depression” (BDI total score > 28) were included in the study. Participants were approached individually and asked to participate in a study on genetic, individual and cultural factors in the interaction of parental rejection during childhood and OXTR polymorphisms in the regulation of depression. Written informed consent was obtained from participants before the start of the experiment. Participants were then brought to the laboratory and assessed through questionnaires and a non-invasive DNA genotyping procedure. All measures were administered in the native language of participants (Italian or Japanese). The whole procedure lasted about 25 min.

### 2.3. Measures

#### 2.3.1. Sociodemographic Questionnaire

A brief demographic questionnaire was administered to obtain information on participants’ age, gender and level of education.

#### 2.3.2. Parental Acceptance-Rejection Questionnaire

Participants completed the short forms for the mother and father versions of the Adult Parental Acceptance-Rejection Questionnaire (Adult PARQ; [66,67,68]). The Adult PARQ assesses the recall of maternal and paternal acceptance–rejection during childhood. The questionnaire consists of 24 items that include key items as appropriate for both mothers and fathers, measuring four sub-scales: (i) warmth/affection: (e.g., “My [mother/father] makes me feel wanted and needed.”); (ii) hostility/aggression (e.g., “My [mother/father] treated me harshly.”); (iii) indifference/neglect (e.g., “My [mother/father] paid no attention to me as long as I did nothing to bother [her/him].”) and (iv) undifferentiated rejection (e.g., “My [mother/father] saw me as a big nuisance.”). Participants indicated how well each statement described their remembrance of their parents’ behaviour in their childhood using a 4-point Likert scale (from “always true” = 4; to “almost never true” = 1). Scores on the PARQ (short form) range from a low of 24 (maximum perceived parental acceptance) to a high of 96 (maximum perceived parental rejection). Scores at or above 60 indicate the perception of qualitatively more rejection than acceptance. The scales showed adequate reliability in both versions (mother and father) and in both languages (Italian and Japanese) with Cronbach alphas > 0.75. For each parent, a single measure of the parental rejection was obtained by summing the total scores of each subscale.

#### 2.3.3. Beck’s Depression Inventory

Beck’s Depression Inventory (BDI [69]) is a 21-item self-report questionnaire used to assess depression severity. The scale measure two dimensions: (1) cognitive/affective (e.g., “I feel the future is hopeless.”); (2) somatic/performance (e.g., “I can’t do any work at all.”). The standardized cut-offs used in the literature recommend that a total score > 28 indicates “severe depression”. Both dimensions had adequate reliability with Cronbach alphas > 0.75 in both languages (Italian and Japanese). For each participant, a single measure of depression was computed by summing the total scores of each dimension.

#### 2.3.4. DNA Genotyping

DNA was extracted from the buccal epithelial cells of the participants and evaluated for the OXTR SNPs rs53576 and rs2254298. In both samples, genomic DNA was extracted using the DNA purification kit QIAamp DNA Mini kit (Qiagen Inc., Tokyo, Japan). The OXTR SNP markers rs53576 and rs2254298 were genotyped using Light-Cycler 480 Real-Time PCR Instrument (Roche Ltd., Basel, Switzerland). Polymerase chain reactions were performed in 10 lL reaction volumes in 84-well plates and contained 10 ng of DNA. Thermal cycler conditions were: (i) 95 °C for 10 min and 40 cycles of 95 °C for 10 s, (ii) 60 °C for 1 min and 72 °C for 1 s and (iii) 40 °C for 30 s. For quality control, a random subset of the sample (about 10%) was reanalysed and was concordant with initial results.

Genotype frequencies among Italian sample were as follows: GG = 32 (41%), AA = 7 (9%) and AG = 39 (50%) for the rs53576 marker; GG = 55 (70.5%) and AG = 23 (29.5%) for the rs2254298 marker. Genotype frequencies among Japanese sample were as follows: GG = 6 (10.9%), AA = 19 (34.5%) and AG = 30 (54.5%) for the rs53576 marker; GG = 27 (49%), AA = 6 (11%) and AG = 22 (40%) for the rs2254298 marker.

In line with previous observed data, a significant association between population and genotype frequencies was found in both OXTR SNPs, *χ*^2^(2) = 21.2, *p* < 0.001 and *χ*^2^(2) = 12.0, *p* = 0.003, for rs53576 and rs2254298, respectively. Similar distributions to previous data indicating a higher A allelic frequency in Asians compared to Caucasians were found for both OXTR SNPs [56,58,59]. For both OXTR SNPs, there was a higher number of G/G homozygotes compared to A/A homozygotes in the Italian sample.

### 2.4. Data Analysis

Preliminary descriptive analyses were executed to investigate missing values and variables’ distributions. Univariate distributions of observed variables were examined for normality [70]. These analyses indicated that there were no missing values or normality problems.

Data were investigated separately for maternal and paternal rejection. Preliminary analyses were conducted to compare maternal and paternal rejection across the two populations. Then, four hierarchical multiple regressions were conducted to investigate the effects of the following factors on depression: (i) parental rejection during childhood, (ii) OXTR SNP rs53576 or rs2254298 genotype, (iii) ethnicity (Caucasian represented by the Italian sample; Asian represented by the Japanese sample) and (iv) interactions between (i), (ii) and (iii). For each regression, predictors were entered in three blocks as follows: (i) parental rejection (maternal or paternal; z-scores), OXTR SNP genotype (rs53576 or rs2254298; dummy coded as follows: GG = 1; AA/AG = 0) and population (dummy coded as follows: Italian = 0; Japanese = 1), (ii) two-way interactions between the variables and (iii) three-way interactions between the variables. When significant, the interaction effects were investigated by applying simple slope analysis and Johnson and Neyman’s (JN) approach [71] to define the lower and upper values of the moderator for which the effect of the predictor on the dependent variable was significant. All analyses were performed with the psych [72] and interaction [73] packages implemented in R 3.6.1 software [74].

## 3. Results

### 3.1. Maternal Rejection

The results of the comparison between the two populations showed a significant difference in the recollection of the degree of maternal rejection, *t*(131) = −3.652, *p* < 0.001, with Italians reporting less maternal rejection, *M* = 95.3, than the Japanese, *M* = 109.7 (see Table 1). Results from the hierarchical regression analysis predicting depression are reported in Table 2. For rs53576, maternal rejection, OXTR gene polymorphism and ethnicity did not significantly predict depression. None of the steps including two-way or three-way interactions were significant. Therefore, the significance test of single parameters was not considered to avoid an increase in the type I error.

For rs2254298, maternal rejection, OXTR gene polymorphism and ethnicity did not significantly predict depression. None of the two-way interactions significantly predicted depression either. The three-way interaction between maternal rejection, OXTR gene polymorphism and ethnicity significantly predicted depression, where ethnicity moderated the interaction effect between maternal rejection and rs2254298 genotype on predicting depression, *R*^2^_diff_ = 0.041, *p* = 0.018.

**Figure 1:**
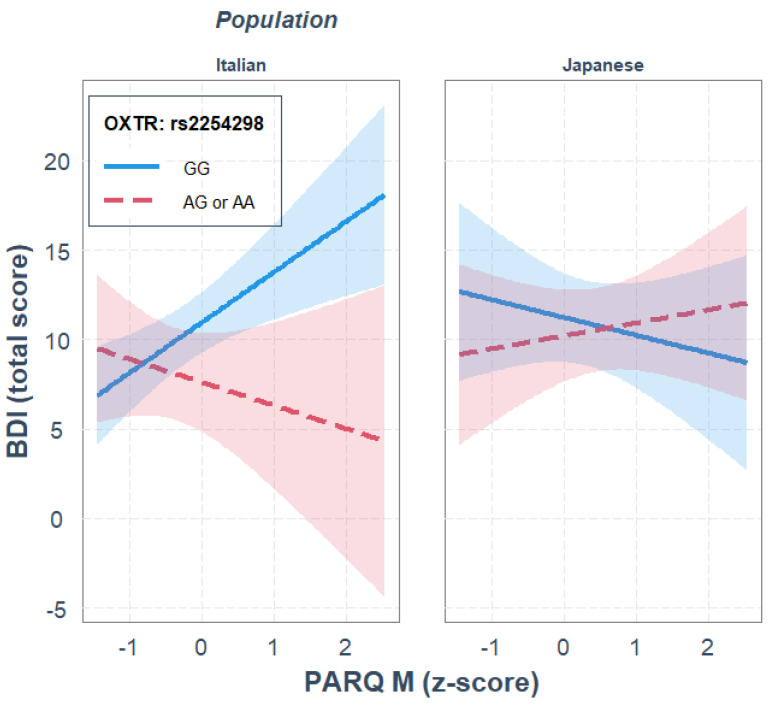
Single regression analyses predicting depression (BDI) from remembered maternal rejection as a function of OXTR rs2254298 gene polymorphism and population.

Parameter analysis of the final model and the JN analysis revealed that, independently of the other variables in the model, GG homozygotes showed higher depression scores than the A carriers, *b* = 3.329, *p* = 0.0043, and that in the Italian sample, only GG homozygotes showed a significant positive association between maternal rejection and BDI scores, *b* = 2.814, *p* = 0.002; see Figure 1. Moreover, the JN analysis indicated that genotype differences were significant when maternal rejection was higher than −0.03 *SD* from the mean, thus supporting the diathesis–stress interpretation. This gene–environment interaction was not observed in the Japanese sample.

### 3.2. Paternal Rejection

The results of the comparison between the two populations showed a significant difference in the recollection of the degree of paternal rejection, *t*(131) = −3.978, *p* < 0.001, with Italians reporting less paternal rejection, *M* = 103.4, than the Japanese, *M* = 122.6 (see Table 1). The results of the hierarchical regression analysis predicting depression are reported in Table 3.

**Figure 2:**
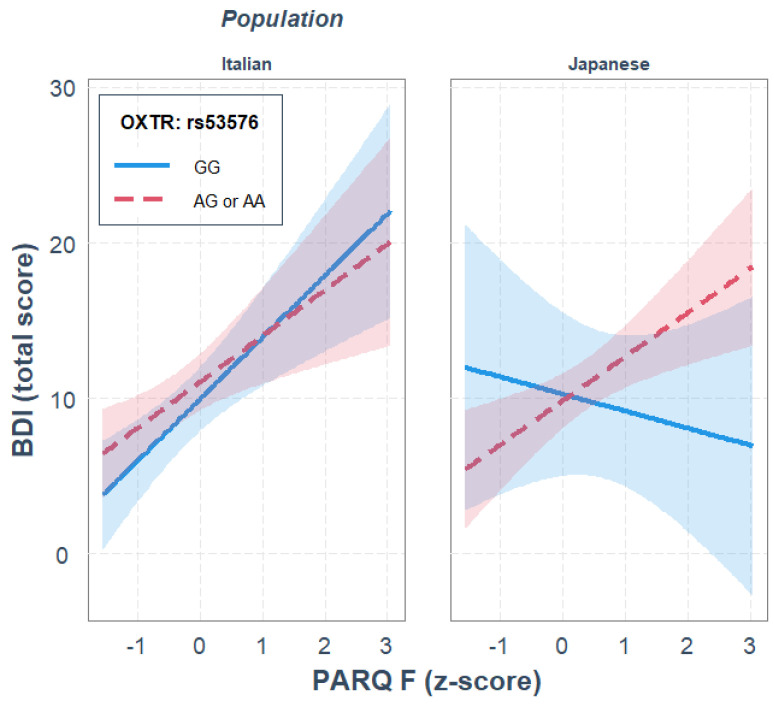
Single regression analyses predicting depression (BDI) from remembered paternal rejection as a function of OXTR rs53576 gene polymorphisms and population.

For rs53576, paternal rejection was a significant predictor of depression, *R*^2^_diff_ = 0.184, *p* < 0.001, while OXTR gene polymorphism and ethnicity did not significantly predict depression. None of the two-way interactions significantly predicted depression. The three-way interaction between paternal rejection, OXTR gene polymorphism and ethnicity significantly predicted depression, where the rs53576 genotype and ethnicity moderated the association between paternal rejection and depression, *R*^2^_diff_ = 0.037, *p* = 0.046. Parameter analysis of the final model revealed that, independently of the other variables in the model, a significant positive association between paternal rejection and BDI scores was observed, *b* = 2.958, *p* = 0.003, and that only in the Japanese sample, and only for A allele carriers, there was a significant positive association between paternal rejection and depression, *b* = 2.830, *p* = 0.002; see Figure 2. Moreover, the JN analysis indicated that genotype differences were significant when the paternal rejection was higher than 2.00 *SD* from the mean, thus supporting the diathesis–stress interpretation. The gene–environment interaction was not observed in the Italian sample.

**Figure 3:**
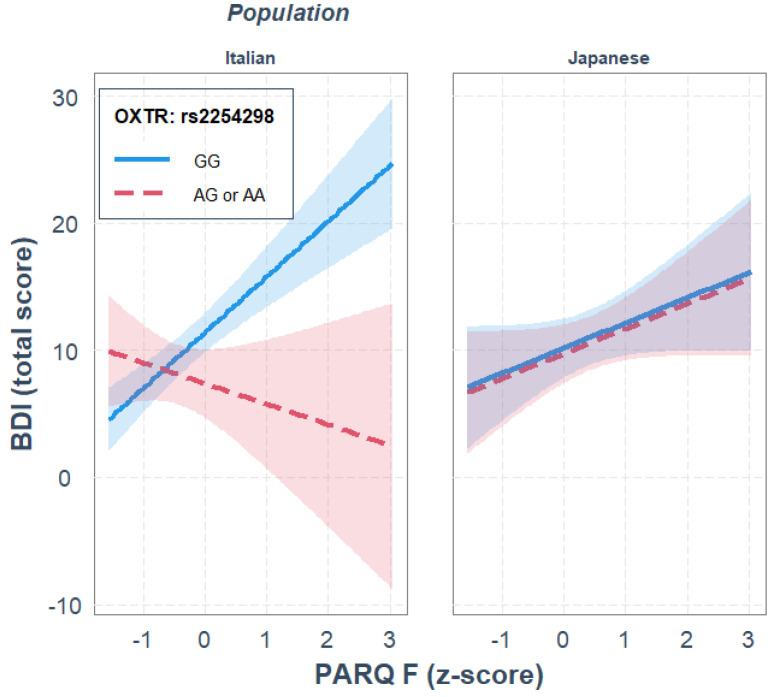
Single regression analyses predicting depression (BDI) from remembered paternal rejection as a function of OXTR rs2254298 gene polymorphisms and population.

For rs2254298, paternal rejection was a significant predictor of depression, *R*^2^_diff_ = 0.183, *p* < 0.001, while OXTR gene polymorphism and ethnicity did not significantly predict depression. None of the two-way interactions significantly predicted depression, *R*^2^_diff_ = 0.042, *p* = 0.086. The three-way interaction between paternal rejection, OXTR gene polymorphism and ethnicity significantly predicted depression, where ethnicity moderated the interaction of paternal rejection and rs2254298 genotype in predicting depression, *R*^2^_diff_ = 0.037, *p* = 0.013. Parameter analysis of the final model revealed that, independently of the other variables in the model, GG homozygotes showed higher depression scores than A carriers, *b* = 3.994, *p* = 0.012, and that only in the Italian sample, and only for GG homozygotes, there was a positive association between paternal rejection and depression, *b* = 4.379, *p* < 0.001; see Figure 3.

Moreover, the JN analysis indicated that genotype differences were significant when paternal rejection was outside the interval [−1.46, −0.19] *SD* from the mean, thus supporting the differential susceptibility interpretation. This gene–environment interaction was not observed in the Japanese sample.

## 4. Discussion

The present study aimed to investigate gene–environment interaction effects between parental rejection and OXTR SNP genotypes of rs53576 and rs2254298 on self-reported depression in adults in different cultural contexts. Here, we examined Italian and Japanese adults and proposed that the nature of gene–environment interactions would differ between these two groups. We considered these two populations because it has been hypothesised that the effects of OXTR genotypes may be moderated by ethnicity and culture, given that allelic frequencies of the OXTR gene differ across countries [56,58,59]. Indeed, this study also confirmed a higher A allelic frequency in Asians compared to Caucasians for both OXTR SNPs.

### 4.1. Parental Rejection

Paternal rejection was found to be a significant and specific predictor of BDI scores, whereas maternal rejection did not significantly predict BDI scores. These findings are in line with previous studies that found love-related behaviours and the influence of fathers to be equally, even significantly, more influential in the development of depression and other psychological problems [75,76] as well as behaviour problems [77] associated with maternal behaviours. In addition, the interaction between parental rejection and ethnicity was non-significant, which is consistent with the universalist perspective asserted in IPARTheory that parental acceptance–rejection is panculturally related to psychological adjustment [12,78,79]. Finally, it is important to note that this latter effect is observed independently of the differences in maternal and paternal rejection scores found in the two populations.

### 4.2. Effect of Ethnicity on Gene–Environment Interactions

The results of the present study indicated that there was a significant three-way interaction between parental rejection, OXTR genotype and ethnicity, supporting the main hypothesis of the study.

Firstly, for rs53576, a significant gene–environment interaction effect was found between paternal rejection and OXTR SNP genotype on depression but only in the Japanese sample. Among A allele carriers, as paternal rejection increased, there was an increase in BDI scores, indicating greater self-reported depression. This finding aligns with the results of a previous study conducted in a Japanese sample suggesting that A alleles for rs53576 may be a risk factor for depressive symptoms among Japanese people [80] and are more congruent with the diathesis–stress interpretation, thus indicating that perceived paternal rejection in those with this polymorphism has more negative effects on depression. In addition, the fact that this gene–environment interaction was only observed in the Japanese sample also dovetails with findings from [58], where the association of the A allele with depression was mediated by collectivistic cultural values, and seems to indicate that this target gene has a higher relevance in the Asian population than in the Caucasian population.

Secondly, for rs2254298, a significant gene–environment interaction effect was found between both paternal and maternal rejection and OXTR SNP genotypes on depression but only in the Italian sample. Among GG homozygotes, as parental rejection increased, there was an increase in BDI scores, indicating greater self-reported depression. Interestingly, this association was the same for both paternal and maternal rejection and was more congruent with the differential susceptibility interpretation, thus indicating that the quality of interpersonal relationships with parents (both mother and father) has a critical effect on depression: it increases the risk of depression if the relationship is perceived as negative or rejecting, whereas it reduces the risk of depression if the relationship is perceived as positive or accepting, thus indicating that the quality of interpersonal relationships with parents (both mother and father) has a critical effect on depression. These findings are in line with previously observed biological differences, such as lower plasma oxytocin levels in GG homozygotes [81] and results reported by Costa et al. [40] in an Italian sample, which showed a positive association between the GG genotype and unipolar depression, and seem to indicate that this target gene has a higher relevance in the Caucasian population than in the Asian population.

### 4.3. Implications

The significant gene–environment interactions found in the present study are a clear indication that further investigation is necessary to better understand and define the effects of parental rejection on psychological adjustment in adulthood, as well as factors underlying individual differences in resilience towards interpersonal rejection. The findings highlight the importance of considering possible environmental influences in behaviour genetics studies and the possibility that contradictory findings on genetic influences on depression could be explained by such environmental factors [82]. Examining both genetic and environmental factors could facilitate the identification of individuals with genetic predispositions at risk for psychopathology. An emerging field, epigenetics, has been proposed to provide a biological basis for gene–environment interactions. Epigenetics refers to reversible modifications to the DNA sequence at a chromatin level that are not encoded in the DNA and can influence levels of gene expression [83]. Specifically, environmental factors have been suggested to confer a depression risk through epigenetic modifications to the genome [84]. One such epigenetic modification is DNA methylation, which involves the addition of methyl groups to cytosine residues within cytosine and guanine dinucleotides, termed “CpG”. DNA methylation has been found to be responsive to the environment, indicating gene–environment interactions [85]. A recent study also found differences in OXTR DNA methylation between depressed and non-depressed individuals. Decreased OXTR exon 1 methylation was observed in depressed women compared to non-depressed women, and the OXTR rs53576 genotype was found to moderate this association [86]. Low maternal care during childhood has also been associated with higher OXTR DNA methylation [87]. However, the effect of epigenetics of OXTR on social and emotional behaviour in human populations is still a relatively new field of inquiry pending firmly conclusive evidence [88].

The significant three-way interaction in the present study also contributes to the growing evidence of gene–culture interactions, which highlights the importance of taking ethnicity into consideration for behaviour genetics studies. In addition, the levels of parental rejection and parenting styles differ across cultures [89], suggesting that interaction effects between parental rejection and OXTR SNP genotypes on depression may differ across cultures and that, depending on the population, there may be different target genes to consider [82]. Hence, the generalisability of the results across cultures should be investigated by replicating the present study across more cultures and considering a broader spectrum of target genes.

### 4.4. Limitations and Future Directions

Some limitations of this study should be acknowledged. Firstly, self-report measures were used, and the study was conducted in a non-clinical sample. Longitudinal studies, as well as the replication of this study with clinical population samples (i.e., individuals clinically diagnosed with depression), would provide a clearer picture of the association between parental rejection and OXTR genotypes in depression during development. 

Secondly, while associations were found between parental rejection, OXTR SNP genotype and ethnicity in depression, the mechanisms mediating this relationship have yet to be elucidated. Future studies can aim to investigate (i) biological markers such as plasma oxytocin levels and (ii) behavioural markers, such as emotion-related behavioural tendencies studied in Kim et al. [63,64], to determine significant mediators through mediation analyses.

Another limitation to mention concerns the size of the samples in the subgroups, which is relatively small, although sufficient to detect significant effects. This latter aspect, together with the replication of data in two different samples, seems to indicate the adequate validity of this study. However, future studies should verify the replicability of these effects by considering larger samples from different populations.

Linked to the latter, another limitation to mention concerns the sampling procedure adopted in the present study. In the present study, participants were selected with quota sampling, which is a non-random sampling technique that was used to create equivalent and balanced samples across the two populations. This led to the selection of participants aged between 18 and 27 years matched by sex across the two populations. Although this had the advantage of creating comparable samples, it may have threatened the validity of the study, as random sampling is always preferable to obtain a representative sample, while the narrow age range considered may have limited the generalisability of the results to the more general adult population. Future studies should replicate the findings on a randomly selected sample that includes adults with a wider age range.

## 5. Conclusions

These findings support the interpersonal acceptance–rejection theory’s predictions about the relationship between perceived acceptance–rejection and psychological adjustment, and they point to the need for future research to take a multicultural and multilevel approach (person-in-context perspective) to better understand why, in some individuals, the effects of parental rejection persist into adulthood.

## Figures and Tables

**Table 1:** Demographic and descriptive data across populations (*N* = 133).

Variable, Statistics	Population		Test
	Italian	Japanese	
*N*	78	55	
Males, *N* (%)	39 (50)	26 (47.3)	
Age, *M* (*SD*)	22.5 (2.2)	19.4 (1.9)	
PARQ Mother, *M* (*SD*)	95.3 (22.0)	109.7 (23.0)	*t*(131) = −3.652 ***
PARQ Father, *M* (*SD*)	103.4 (26.8)	122.6 (28.0)	*t*(131) = −3.978 ***
BDI, *M* (*SD*)	9.7 (6.8)	10.7 (5.9)	*t*(131) = −0.886
Clinical depression, *N*	0	0	

*Note*. PARQ Mother = maternal form of the Adult Parental Acceptance-Rejection Questionnaire; PARQ Father = paternal form of the Adult Parental Acceptance-Rejection Questionnaire; BDI = total score on the Beck’s Depression Inventory; Clinical depression = participants with a total score on BDI > 28; *** *p* < 0.001.

**Table 2:** Hierarchical multiple regression analyses predicting depression (BDI) from remembered maternal rejection, OXTR gene polymorphism, population and their interaction.

Predictor	SNPs					
	rs53576			rs2254298		
	*R* ^2^ *_diff_*	*b*	*β*	*R* ^2^ *_diff_*	*b*	*β*
Step 1	0.032			0.039		
PARQ M		1.0	0.157		1.0	0.152
Pop		0.1	0.005		0.7	0.055
OXTR		−1.0	−0.073		1.5	0.110
Step 2	0.030			0.029		
PARQ M		2.7 *	0.422 *		0.8	0.129
Pop		0.0	0.000		2.5	0.195
OXTR		−1.4	−0.096		2.5	0.189
PARQ M × OXTR		−1.5	−0.144		1.2	0.149
PARQ M × Pop		−2.6 *	−0.262 *		−1.5	−0.154
Pop × OXTR		0.8	0.027		−2.6	−0.163
Step 3	0.008			0.041 *		
PARQ M		2.2	0.345		−1.3	−0.203
Pop		0.0	0.002		2.6	0.199
OXTR		−1.2	−0.082		3.3 *	0.253 *
PARQ M × OXTR		−0.6	−0.061		4.1 *	0.497 *
PARQ M × Pop		−1.7	−0.177		2.0	0.207
Pop × OXTR		1.8	0.058		−2.3	−0.144
PARQ M × OXTR × Pop		−3.1	−0.129		−5.8 *	−0.409 *
Total R^2^	0.070			0.109 *		

*Note*. *N* = 133. SNPs = single-nucleotide polymorphisms; PARQ M = maternal form of the Adult Parental Acceptance-Rejection Questionnaire; Pop = population dummy coding (Italian = 0; Japanese = 1); OXTR = oxytocin receptor polymorphism dummy coding (AG or AA = 0; GG = 1). * *p* < 0.05.

**Table 3:** Hierarchical multiple regression analyses predicting depression (BDI) from remembered paternal rejection, OXTR gene polymorphism, population and their interaction.

Predictor	SNPs					
	rs53576			rs2254298		
	*R* ^2^ *_diff_*	*b*	*β*	*R* ^2^ *_diff_*	*b*	*β*
Step 1	0.184 ***			0.183 ***		
PARQ F		2.8 ***	0.444 ***		2.7 ***	0.431 ***
Pop		−1.3	−0.102		−0.6	−0.044
OXTR		−1.5	−0.103		1.2	0.094
Step 2	0.013			0.042		
PARQ F		3.7 ***	0.587 ***		1.1	0.180
Pop		−1.2	−0.095		1.8	0.135
OXTR		−1.5	−0.106		2.8	0.209
PARQ F × OXTR		−0.6	−0.058		2.6 *	0.334 *
PARQ F × Pop		−1.6	−0.161		−0.5	−0.049
Pop × OXTR		−0.1	−0.002		−3.4	−0.210
Step 3	0.025 *			0.037 *		
PARQ F		3.0 **	0.466 **		−1.6	−0.255
Pop		−1.2	−0.093		2.3	0.179
OXTR		−1.1	−0.077		4.0 *	0.303 *
PARQ F × OXTR		1.0	0.093		6.0 ***	0.763 ***
PARQ F × Pop		−0.1	−0.013		3.6	0.367
Pop × OXTR		1.5	0.050		−3.6	−0.223
PARQ F × OXTR × Pop		−4.9 *	−0.243 *		−6.0 *	−0.437 *
Total R^2^	0.222 ***			0.262 ***		

*Note*. *N* = 133. SNPs = single-nucleotide polymorphisms; PARQ F = paternal form of the Adult Parental Acceptance-Rejection Questionnaire; Pop = population dummy coding (Italian = 0; Japanese = 1); OXTR = oxytocin receptor polymorphisms dummy coding (AG or AA = 0; GG = 1). * *p* < 0.05; ** *p* < 0.01; *** *p* < 0.001.

## Data Availability

The dataset that supports the findings of this study and all other materials are available from the corresponding author upon reasonable request.
